# Supplementary material for: Knowledge, Awareness, and Attitude of Healthcare Stakeholders on Alzheimer’s Disease and Dementia in Qatar
Source: Int J Environ Res Public Health. 2023 Mar 3;20(5):4535. doi: 10.3390/ijerph20054535 (PMC10002196; doi:10.3390/ijerph20054535)
Supplement: Supplementary file 1 [file ijerph-20-04535-s001.zip › ijerph-2211040-supplementary.pdf]

Knowledge, Awareness, and Attitude of Healthcare Stakeholders on Alzheimer's Disease and Dementia in Qatar

Pradipta Paul[1], Ziyad Riyad Mahfoud[1,2], Rayaz Malik[3,4,5], Ridhima Kaul[1], Phyllis Sui Muffuh Navti[6], Deema Al-Sheikhly[1,6] and Ali Chaari[7,*]

[1]Department of Medical Education, Weill Cornell Medicine-Qatar, Doha, Qatar
[2]Division of Epidemiology, Department of Population Health Sciences, Weill Cornell Medicine, NY, USA
[3]Department of Medicine, Weill Cornell Medicine-Qatar, Doha, Qatar
[4]Faculty of Biology, Medicine and Health, University of Manchester, Manchester, UK
[5]Faculty of Science and Engineering, Manchester Metropolitan University, Manchester, UK
[6]Division of Continuing Professional Development, Weill Cornell Medicine-Qatar, Doha, Qatar
[7]Premedical Division, Weill Cornell Medicine-Qatar, Doha, Qatar

***Corresponding Author:**
Ali Chaari, PhD, Assistant Professor of Biology, Weill Cornell Medicine-Qatar, Doha, Qatar; E-mail: alc2033@qatar-med.cornell.edu, ali.chaari@yahoo.fr, Tel.: +974 4492 8432

Demographic information

Please indicate your age (years).

- 17-20
- 21-30
- 31-40
- 41-50
- 51-60
- 61-70
- Above 70

What best describes your current occupation in healthcare?

- Physician
- Nurse
- Dentist
- Pharmacist
- Social Worker
- Allied Health Professional
- Student
- Administrator
- Researcher
- Educator
- Insurance Representative
- Other (please specify) ____

In which country is your primary location of practice/work? [COUNTRIES LIST DROPDOWN]

What approximate % of your current patients are elderly (>60 years of age)

- 0-10
- 11-30
- 31-50
- 51-70
- 71-100

How much exposure have you had to dementia or neurodegenerative disease patients in a healthcare environment?

- Never
- Very Rarely (<10 patients/year)
- Rarely (11-50 patients/year)
- Occasionally (51-200 patients/year)
- Frequently (201-350 patients/ year)
- Very Frequently (>350 patients/year)

Have you had any training in dementia/neurodegenerative disease in the last 2 years? This may include webinars, training courses or grand rounds. (Yes/No)

Would you be interested in a webinar series that aims to increase your knowledge and competence with regards to patients with neurodegenerative disease and its disorders?

- Yes
- No

How much do you agree with the following statements?

**QUESTION NUMBER & ITEM**	Strongly Disagree	Disagree	Neutral	Agree	Strongly Agree
1. The loss of memory and forgetting names, appointments, and task repetition of questions are normal in the elderly and do not require medical consultation					
2. A change in the planning matters of everyday life and difficulty in balancing financial accounts is expected in the elderly					
3. If your relative was diagnosed with Alzheimer's disease, you would prefer not to tell the person with the illness.					
4. It is best for a patient diagnosed with Alzheimer's to avoid going to social events and live activities to avoid embarrassment to the patient					
5. Alzheimer's disease may result from black magic or psychological distress or bad eye					
6. In the case of appearance of symptoms of dementia and memory disorder for a relative, you would resort to popular types of alternative medicine					
7. In the case of difficulty in performing everyday tasks in a patient with Alzheimer's, it is necessary to resort to the judiciary to save the patient's rights					

8. You would feel embarrassed if one of your close relatives was diagnosed with Alzheimer's disease?					
9. You would tend to deny the diagnosis of Alzheimer's disease in one of your relatives?					
10. Patients with Alzheimer's disease should be looked after by the state in nursing homes and not at home?					
11. Dementia research and innovation can greatly improve the outlook for dementia patients, their family, and their providers					
12. The earliest diagnosis of Alzheimer's disease can be helpful to start the treatment earlier and to have a better response to the treatment					
13. Artificial Intelligence has a potential to be of service for the diagnosis, treatment and management of neurodegenerative diseases and related disorders such as dementia					
14. Healthcare professionals from most if not all fields should be aware of the most recent updates in the field of neurodegenerative disease and its disorders					
15. Lifestyle shapes the brain					
16. There are associations between physical activity, sleep and cognitive function in older adults					

Are you aware of the following?

**QUESTION NUMBER & ITEM**	Not at all aware	Slightly aware	Somewhat Aware	Moderately aware	Extremely aware
1. The mechanisms behind neurodegenerative diseases like Alzheimer's, Parkinson's, Huntington's					
2. The biochemical significance of protein misfolding and amyloid formation					
3. Artificial Intelligence and its applications in daily life					
4. Artificial Intelligence and its applications in healthcare for diagnosis and disease detection					
5. Corneal Confocal Microscopy and its potential to be used in diagnosis of peripheral neuropathies and central neurodegenerative diseases compared to traditional aspects like symptoms/signs, quantitative sensory testing, nerve.					

Composition of WCM-Q CPD mailing List

**Total Contacts in Mailing List**	**10,307**
**Total Contacts with Known Profession**	**4,085 (39.6%)**
Physicians	764 (18.7%)
Nurses	2,440 (59.7%)
Allied health Professionals	190 (4.6%)
Pharmacists	277 (6.7%
Dentists	135 (3.3%)
Researchers	25 (0.61%)
Educators	14 (0.34%)
Students	191 (4.7%)
Others	49 (1.2%)
**Total Contacts with Known Country of Origin**	**4,085 (39.6%)**
Australia	3
Bahrain	1
Brazil	2
Brunei	1
Cambodia	1
Canada	5
Egypt	2
France	1
Greece	1
India	58 (1.42%)
Ireland	1
Jordan	4
Kuwait	2
Lebanon	5
Lesotho	1
Maseru	1

Morocco	1
Oman	2
Pakistan	6
Palestine	1
Pero	1
Philippines	38
Qatar	3,720 (91.1%)
Rwanda	2
Saudi Arabia	19 (0.46%)
Sri Lanka	1
Sudan	6
Taiwan	1
Thailand	2
Tunisia	1
UAE	10 (0.24%)
United Kingdom	3
USA	182 (4.5%)

Note: This list has been updated in February 2023, which is why the total contacts of health professionals (10307) is higher than what was when the survey was distributed (9742) in April-May 2022; however, this increase is small, and the proportion of professionals are likely similar to during study period. Of the current 10,307 contacts, profession, and country of origin (based on de-identified data from email IP addresses) is available for only 4,085, although all are healthcare professionals or stakeholders. These data were retrieved from MailChimp.
